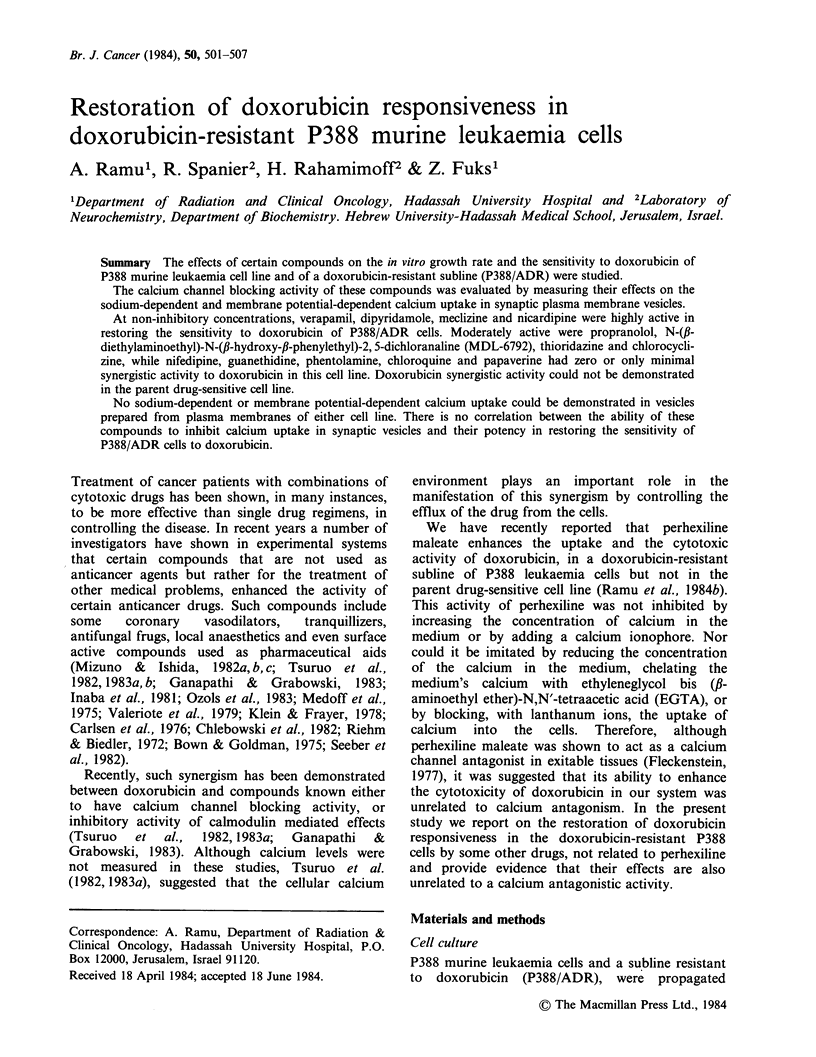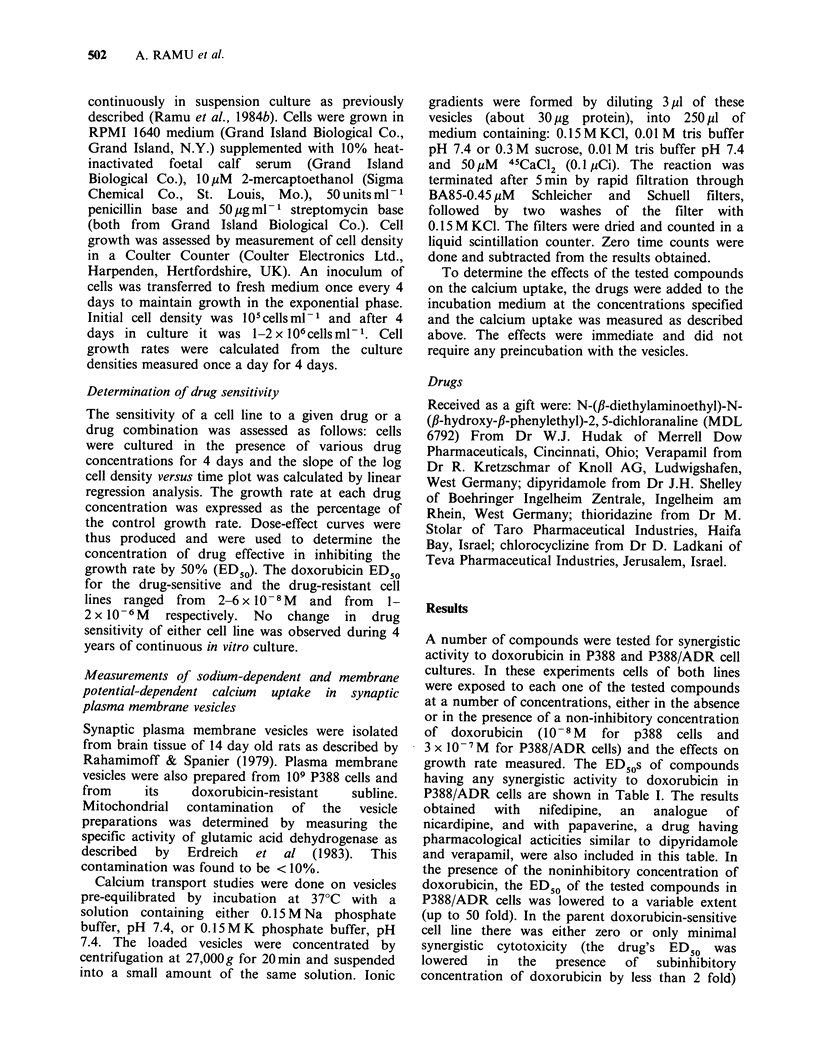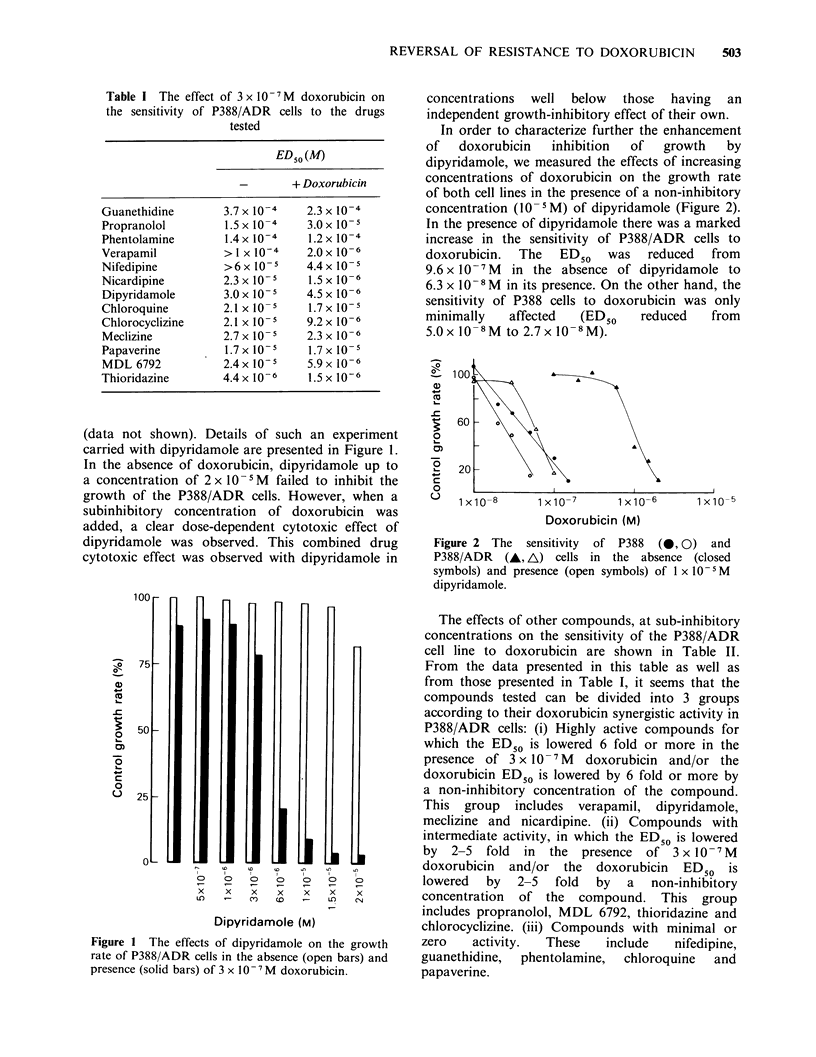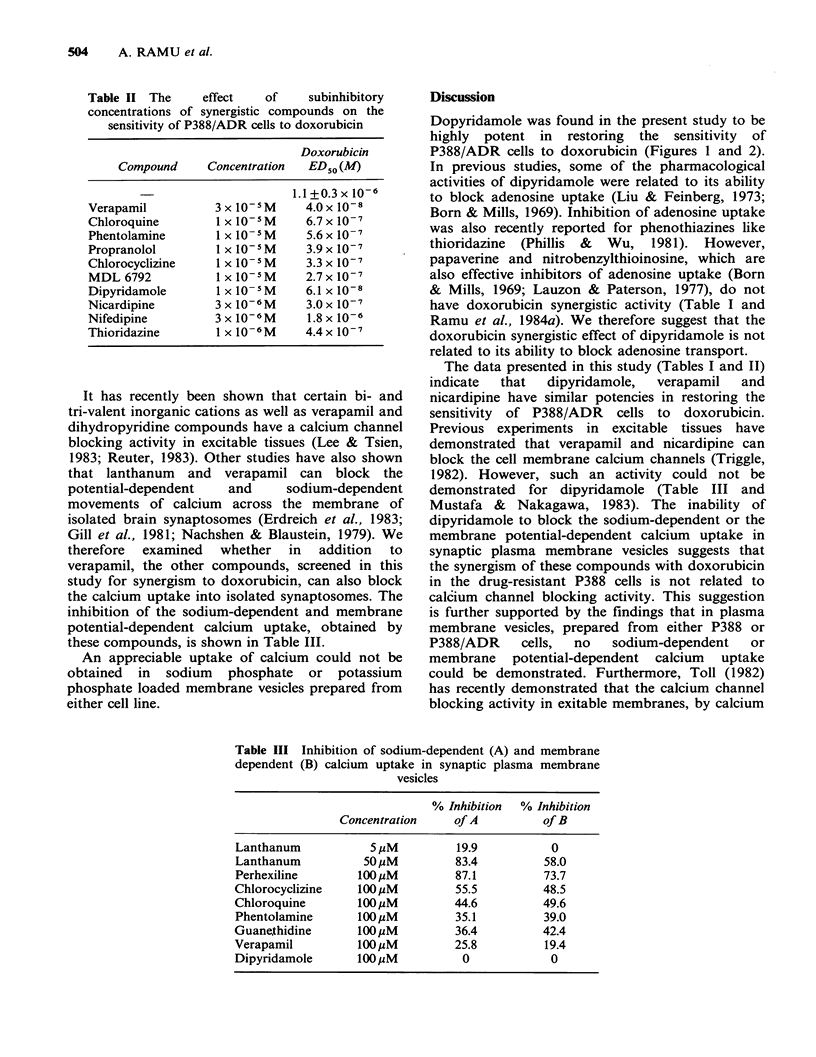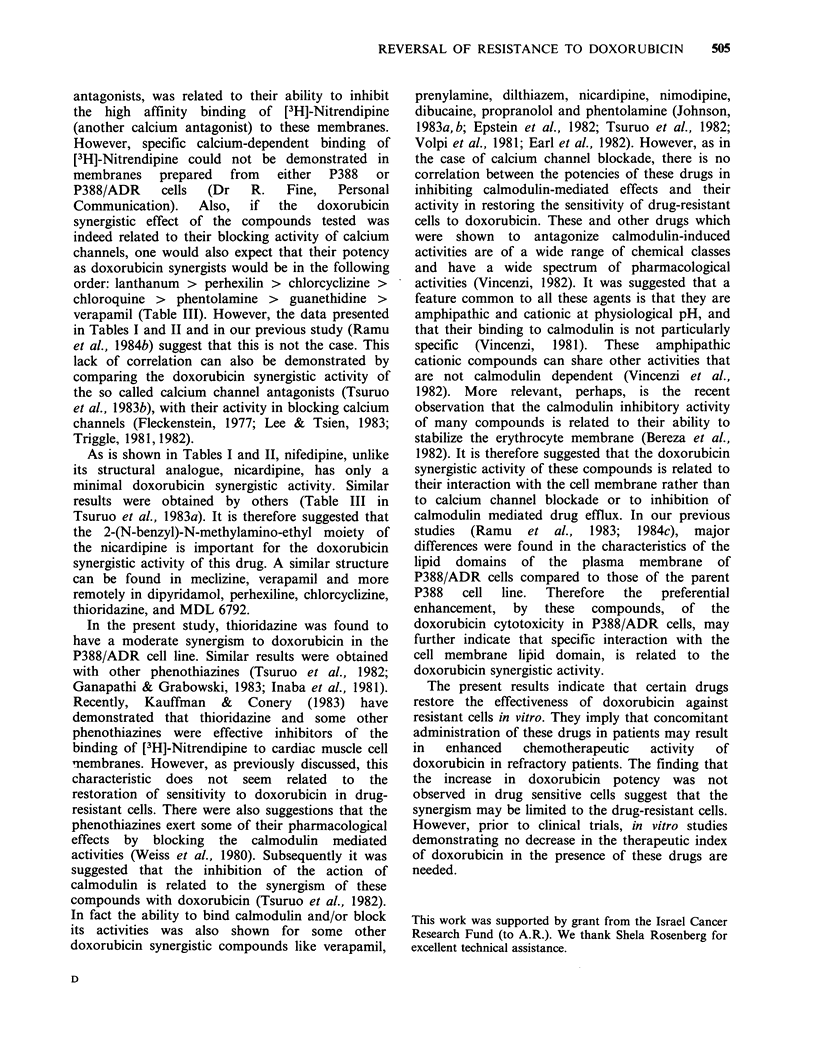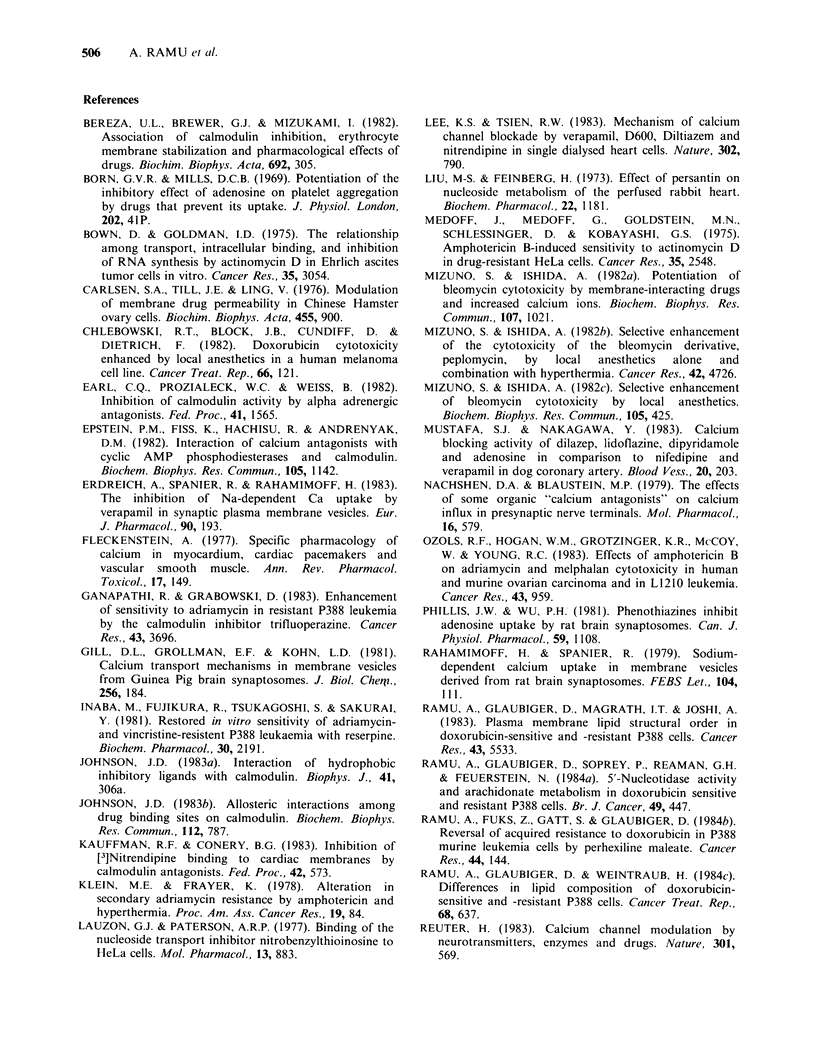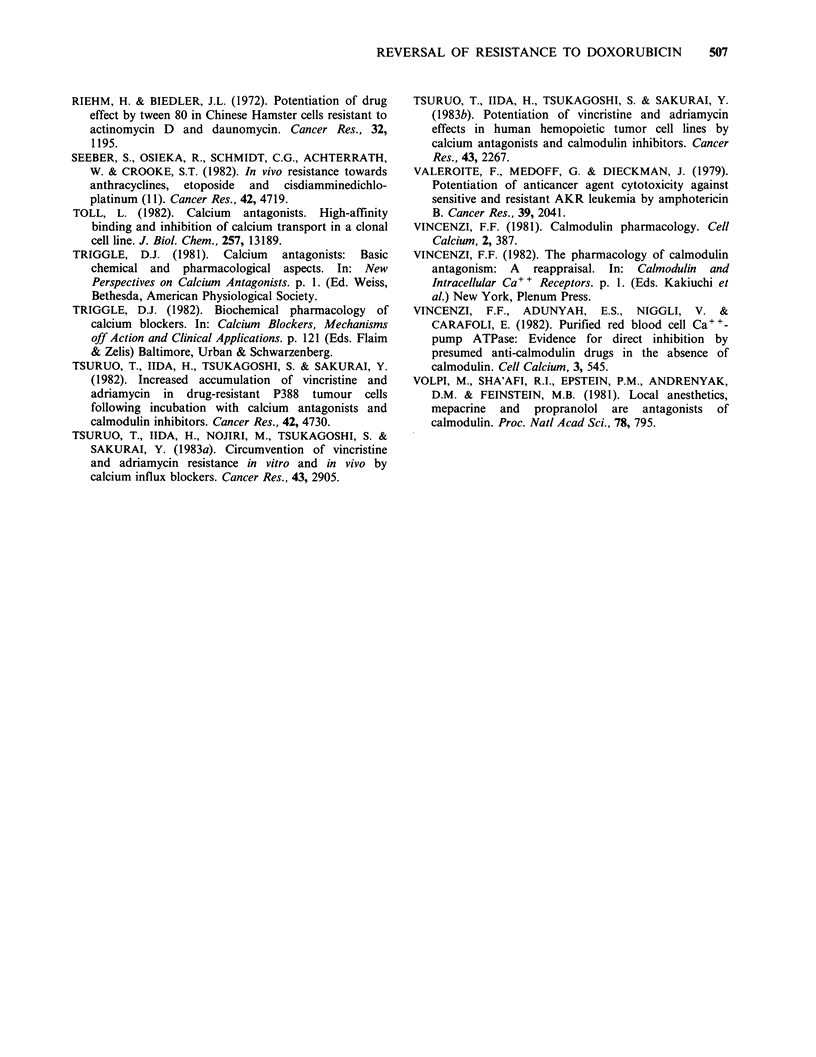# Restoration of doxorubicin responsiveness in doxorubicin-resistant P388 murine leukaemia cells.

**DOI:** 10.1038/bjc.1984.207

**Published:** 1984-10

**Authors:** A. Ramu, R. Spanier, H. Rahamimoff, Z. Fuks

## Abstract

The effects of certain compounds on the in vitro growth rate and the sensitivity to doxorubicin of P388 murine leukaemia cell line and of a doxorubicin-resistant subline (P388/ADR) were studied. The calcium channel blocking activity of these compounds was evaluated by measuring their effects on the sodium-dependent and membrane potential-dependent calcium uptake in synaptic plasma membrane vesicles. At non-inhibitory concentrations, verapamil, dipyridamole, meclizine and nicardipine were highly active in restoring the sensitivity to doxorubicin of P388/ADR cells. Moderately active were propranolol, N-(beta-diethylaminoethyl)-N-(beta-hydroxy-beta-phenylethyl)-2,5-dich loranaline (MDL-6792), thioridazine and chlorocyclizine, while nifedipine, guanethidine, phentolamine, chloroquine and papaverine had zero or only minimal synergistic activity to doxorubicin in this cell line. Doxorubicin synergistic activity could not be demonstrated in the parent drug-sensitive cell line. No sodium-dependent or membrane potential-dependent calcium uptake could be demonstrated in vesicles prepared from plasma membranes of either cell line. There is no correlation between the ability of these compounds to inhibit calcium uptake in synaptic vesicles and their potency in restoring the sensitivity of P388/ADR cells to doxorubicin.


Br. J. Cancer (1984), **50**, 501–507

### Restoration of doxorubicin responsiveness in doxorubicin-resistant P388 murine leukaemia cells

A. Ramu[1], R. Spanier[2], H. Rahamimoff[2] & Z. Fuks[1]

[1]*Department of Radiation and Clinical Oncology, Hadassah University Hospital and* [2]*Laboratory of Neurochemistry, Department of Biochemistry. Hebrew University-Hadassah Medical School, Jerusalem, Israel.*

**Summary** The effects of certain compounds on the *in vitro* growth rate and the sensitivity to doxorubicin of P388 murine leukaemia cell line and of a doxorubicin-resistant subline (P388/ADR) were studied.

The calcium channel blocking activity of these compounds was evaluated by measuring their effects on the sodium-dependent and membrane potential-dependent calcium uptake in synaptic plasma membrane vesicles.

At non-inhibitory concentrations, verapamil, dipyridamole, meclizine and nicardipine were highly active in restoring the sensitivity to doxorubicin of P388/ADR cells. Moderately active were propranolol, N-($\beta$-diethylaminoethyl)-N-($\beta$-hydroxy-$\beta$-phenylethyl)-2, 5-dichloranaline (MDL-6792), thioridazine and chlorocyclizine, while nifedipine, guanethidine, phentolamine, chloroquine and papaverine had zero or only minimal synergistic activity to doxorubicin in this cell line. Doxorubicin synergistic activity could not be demonstrated in the parent drug-sensitive cell line.

No sodium-dependent or membrane potential-dependent calcium uptake could be demonstrated in vesicles prepared from plasma membranes of either cell line. There is no correlation between the ability of these compounds to inhibit calcium uptake in synaptic vesicles and their potency in restoring the sensitivity of P388/ADR cells to doxorubicin.

Treatment of cancer patients with combinations of cytotoxic drugs has been shown, in many instances, to be more effective than single drug regimens, in controlling the disease. In recent years a number of investigators have shown in experimental systems that certain compounds that are not used as anticancer agents but rather for the treatment of other medical problems, enhanced the activity of certain anticancer drugs. Such compounds include some coronary vasodilators, tranquillizers, antifungal frugs, local anaesthetics and even surface active compounds used as pharmaceutical aids (Mizuno & Ishida, 1982*a,b,c*; Tsuruo *et al.*, 1982, 1983*a,b*; Ganapathi & Grabowski, 1983; Inaba *et al.*, 1981; Ozols *et al.*, 1983; Medoff *et al.*, 1975; Valeriote *et al.*, 1979; Klein & Frayer, 1978; Carlsen *et al.*, 1976; Chlebowski *et al.*, 1982; Riehm & Biedler, 1972; Bown & Goldman, 1975; Seeber *et al.*, 1982).

Recently, such synergism has been demonstrated between doxorubicin and compounds known either to have calcium channel blocking activity, or inhibitory activity of calmodulin mediated effects (Tsuruo *et al.*, 1982, 1983*a*; Ganapathi & Grabowski, 1983). Although calcium levels were not measured in these studies, Tsuruo *et al.* (1982, 1983*a*), suggested that the cellular calcium environment plays an important role in the manifestation of this synergism by controlling the efflux of the drug from the cells.

We have recently reported that perhexiline maleate enhances the uptake and the cytotoxic activity of doxorubicin, in a doxorubicin-resistant subline of P388 leukaemia cells but not in the parent drug-sensitive cell line (Ramu *et al.*, 1984*b*). This activity of perhexiline was not inhibited by increasing the concentration of calcium in the medium or by adding a calcium ionophore. Nor could it be imitated by reducing the concentration of the calcium in the medium, chelating the medium's calcium with ethyleneglycol bis ($\beta$-aminoethyl ether)-N,N′-tetraacetic acid (EGTA), or by blocking, with lanthanum ions, the uptake of calcium into the cells. Therefore, although perhexiline maleate was shown to act as a calcium channel antagonist in exitable tissues (Fleckenstein, 1977), it was suggested that its ability to enhance the cytotoxicity of doxorubicin in our system was unrelated to calcium antagonism. In the present study we report on the restoration of doxorubicin responsiveness in the doxorubicin-resistant P388 cells by some other drugs, not related to perhexiline and provide evidence that their effects are also unrelated to a calcium antagonistic activity.

### Materials and methods

### *Cell culture*

P388 murine leukaemia cells and a subline resistant to doxorubicin (P388/ADR), were propagated

Correspondence: A. Ramu, Department of Radiation & Clinical Oncology, Hadassah University Hospital, P.O. Box 12000, Jerusalem, Israel 91120.

Received 18 April 1984; accepted 18 June 1984.

© The Macmillan Press Ltd., 1984

continuously in suspension culture as previously described (Ramu *et al.*, 1984*b*). Cells were grown in RPMI 1640 medium (Grand Island Biological Co., Grand Island, N.Y.) supplemented with 10% heat-inactivated foetal calf serum (Grand Island Biological Co.), 10 $\mu$M 2-mercaptoethanol (Sigma Chemical Co., St. Louis, Mo.), 50 units $ml^{-1}$ penicillin base and 50 $\mu g\,ml^{-1}$ streptomycin base (both from Grand Island Biological Co.). Cell growth was assessed by measurement of cell density in a Coulter Counter (Coulter Electronics Ltd., Harpenden, Hertfordshire, UK). An inoculum of cells was transferred to fresh medium once every 4 days to maintain growth in the exponential phase. Initial cell density was $10^5$ cells $ml^{-1}$ and after 4 days in culture it was $1–2 \times 10^6$ cells $ml^{-1}$. Cell growth rates were calculated from the culture densities measured once a day for 4 days.

### *Determination of drug sensitivity*

The sensitivity of a cell line to a given drug or a drug combination was assessed as follows: cells were cultured in the presence of various drug concentrations for 4 days and the slope of the log cell density *versus* time plot was calculated by linear regression analysis. The growth rate at each drug concentration was expressed as the percentage of the control growth rate. Dose-effect curves were thus produced and were used to determine the concentration of drug effective in inhibiting the growth rate by 50% ($ED_{50}$). The doxorubicin $ED_{50}$ for the drug-sensitive and the drug-resistant cell lines ranged from $2–6 \times 10^{-8}$ M and from $1–2 \times 10^{-6}$ M respectively. No change in drug sensitivity of either cell line was observed during 4 years of continuous *in vitro* culture.

### *Measurements of sodium-dependent and membrane potential-dependent calcium uptake in synaptic plasma membrane vesicles*

Synaptic plasma membrane vesicles were isolated from brain tissue of 14 day old rats as described by Rahamimoff & Spanier (1979). Plasma membrane vesicles were also prepared from $10^9$ P388 cells and from its doxorubicin-resistant subline. Mitochondrial contamination of the vesicle preparations was determined by measuring the specific activity of glutamic acid dehydrogenase as described by Erdreich *et al* (1983). This contamination was found to be <10%.

Calcium transport studies were done on vesicles pre-equilibrated by incubation at 37°C with a solution containing either 0.15 M Na phosphate buffer, pH 7.4, or 0.15 M K phosphate buffer, pH 7.4. The loaded vesicles were concentrated by centrifugation at 27,000 *g* for 20 min and suspended into a small amount of the same solution. Ionic gradients were formed by diluting 3 $\mu$l of these vesicles (about 30 $\mu$g protein), into 250 $\mu$l of medium containing: 0.15 M KCl, 0.01 M tris buffer pH 7.4 or 0.3 M sucrose, 0.01 M tris buffer pH 7.4 and 50 $\mu$M $^{45}CaCl_2$ (0.1 $\mu$Ci). The reaction was terminated after 5 min by rapid filtration through BA85-0.45 $\mu$M Schleicher and Schuell filters, followed by two washes of the filter with 0.15 M KCl. The filters were dried and counted in a liquid scintillation counter. Zero time counts were done and subtracted from the results obtained.

To determine the effects of the tested compounds on the calcium uptake, the drugs were added to the incubation medium at the concentrations specified and the calcium uptake was measured as described above. The effects were immediate and did not require any preincubation with the vesicles.

### *Drugs*

Received as a gift were: N-($\beta$-diethylaminoethyl)-N-($\beta$-hydroxy-$\beta$-phenylethyl)-2, 5-dichloranaline (MDL 6792) From Dr W.J. Hudak of Merrell Dow Pharmaceuticals, Cincinnati, Ohio; Verapamil from Dr R. Kretzschmar of Knoll AG, Ludwigshafen, West Germany; dipyridamole from Dr J.H. Shelley of Boehringer Ingelheim Zentrale, Ingelheim am Rhein, West Germany; thioridazine from Dr M. Stolar of Taro Pharmaceutical Industries, Haifa Bay, Israel; chlorocyclizine from Dr D. Ladkani of Teva Pharmaceutical Industries, Jerusalem, Israel.

### Results

A number of compounds were tested for synergistic activity to doxorubicin in P388 and P388/ADR cell cultures. In these experiments cells of both lines were exposed to each one of the tested compounds at a number of concentrations, either in the absence or in the presence of a non-inhibitory concentration of doxorubicin ($10^{-8}$ M for p388 cells and $3 \times 10^{-7}$ M for P388/ADR cells) and the effects on growth rate measured. The $ED_{50}$s of compounds having any synergistic activity to doxorubicin in P388/ADR cells are shown in Table I. The results obtained with nifedipine, an analogue of nicardipine, and with papaverine, a drug having pharmacological acticities similar to dipyridamole and verapamil, were also included in this table. In the presence of the noninhibitory concentration of doxorubicin, the $ED_{50}$ of the tested compounds in P388/ADR cells was lowered to a variable extent (up to 50 fold). In the parent doxorubicin-sensitive cell line there was either zero or only minimal synergistic cytotoxicity (the drug's $ED_{50}$ was lowered in the presence of subinhibitory concentration of doxorubicin by less than 2 fold)

**Table I** The effect of $3\times10^{-7}$ M doxorubicin on the sensitivity of P388/ADR cells to the drugs tested

| | $ED_{50}(M)$ | |
|---|---|---|
| | – | + *Doxorubicin* |
| Guanethidine | $3.7\times10^{-4}$ | $2.3\times10^{-4}$ |
| Propranolol | $1.5\times10^{-4}$ | $3.0\times10^{-5}$ |
| Phentolamine | $1.4\times10^{-4}$ | $1.2\times10^{-4}$ |
| Verapamil | $>1\times10^{-4}$ | $2.0\times10^{-6}$ |
| Nifedipine | $>6\times10^{-5}$ | $4.4\times10^{-5}$ |
| Nicardipine | $2.3\times10^{-5}$ | $1.5\times10^{-6}$ |
| Dipyridamole | $3.0\times10^{-5}$ | $4.5\times10^{-6}$ |
| Chloroquine | $2.1\times10^{-5}$ | $1.7\times10^{-5}$ |
| Chlorocyclizine | $2.1\times10^{-5}$ | $9.2\times10^{-6}$ |
| Meclizine | $2.7\times10^{-5}$ | $2.3\times10^{-6}$ |
| Papaverine | $1.7\times10^{-5}$ | $1.7\times10^{-5}$ |
| MDL 6792 | $2.4\times10^{-5}$ | $5.9\times10^{-6}$ |
| Thioridazine | $4.4\times10^{-6}$ | $1.5\times10^{-6}$ |

(data not shown). Details of such an experiment carried with dipyridamole are presented in Figure 1. In the absence of doxorubicin, dipyridamole up to a concentration of $2\times10^{-5}$ M failed to inhibit the growth of the P388/ADR cells. However, when a subinhibitory concentration of doxorubicin was added, a clear dose-dependent cytotoxic effect of dipyridamole was observed. This combined drug cytotoxic effect was observed with dipyridamole in

**Figure 1** The effects of dipyridamole on the growth rate of P388/ADR cells in the absence (open bars) and presence (solid bars) of $3\times10^{-7}$ M doxorubicin.

concentrations well below those having an independent growth-inhibitory effect of their own.

In order to characterize further the enhancement of doxorubicin inhibition of growth by dipyridamole, we measured the effects of increasing concentrations of doxorubicin on the growth rate of both cell lines in the presence of a non-inhibitory concentration ($10^{-5}$ M) of dipyridamole (Figure 2). In the presence of dipyridamole there was a marked increase in the sensitivity of P388/ADR cells to doxorubicin. The $ED_{50}$ was reduced from $9.6\times10^{-7}$ M in the absence of dipyridamole to $6.3\times10^{-8}$ M in its presence. On the other hand, the sensitivity of P388 cells to doxorubicin was only minimally affected ($ED_{50}$ reduced from $5.0\times10^{-8}$ M to $2.7\times10^{-8}$ M).

**Figure 2** The sensitivity of P388 (●, ○) and P388/ADR (▲, △) cells in the absence (closed symbols) and presence (open symbols) of $1\times10^{-5}$ M dipyridamole.

The effects of other compounds, at sub-inhibitory concentrations on the sensitivity of the P388/ADR cell line to doxorubicin are shown in Table II. From the data presented in this table as well as from those presented in Table I, it seems that the compounds tested can be divided into 3 groups according to their doxorubicin synergistic activity in P388/ADR cells: (i) Highly active compounds for which the $ED_{50}$ is lowered 6 fold or more in the presence of $3\times10^{-7}$ M doxorubicin and/or the doxorubicin $ED_{50}$ is lowered by 6 fold or more by a non-inhibitory concentration of the compound. This group includes verapamil, dipyridamole, meclizine and nicardipine. (ii) Compounds with intermediate activity, in which the $ED_{50}$ is lowered by 2–5 fold in the presence of $3\times10^{-7}$ M doxorubicin and/or the doxorubicin $ED_{50}$ is lowered by 2–5 fold by a non-inhibitory concentration of the compound. This group includes propranolol, MDL 6792, thioridazine and chlorocyclizine. (iii) Compounds with minimal or zero activity. These include nifedipine, guanethidine, phentolamine, chloroquine and papaverine.

**Table II** The effect of subinhibitory concentrations of synergistic compounds on the sensitivity of P388/ADR cells to doxorubicin

| *Compound* | *Concentration* | *Doxorubicin* $ED_{50}(M)$ |
|---|---|---|
| — | | $1.1 \pm 0.3 \times 10^{-6}$ |
| Verapamil | $3 \times 10^{-5}$ M | $4.0 \times 10^{-8}$ |
| Chloroquine | $1 \times 10^{-5}$ M | $6.7 \times 10^{-7}$ |
| Phentolamine | $1 \times 10^{-5}$ M | $5.6 \times 10^{-7}$ |
| Propranolol | $1 \times 10^{-5}$ M | $3.9 \times 10^{-7}$ |
| Chlorocyclizine | $1 \times 10^{-5}$ M | $3.3 \times 10^{-7}$ |
| MDL 6792 | $1 \times 10^{-5}$ M | $2.7 \times 10^{-7}$ |
| Dipyridamole | $1 \times 10^{-5}$ M | $6.1 \times 10^{-8}$ |
| Nicardipine | $3 \times 10^{-6}$ M | $3.0 \times 10^{-7}$ |
| Nifedipine | $3 \times 10^{-6}$ M | $1.8 \times 10^{-6}$ |
| Thioridazine | $1 \times 10^{-6}$ M | $4.4 \times 10^{-7}$ |

It has recently been shown that certain bi- and tri-valent inorganic cations as well as verapamil and dihydropyridine compounds have a calcium channel blocking activity in excitable tissues (Lee & Tsien, 1983; Reuter, 1983). Other studies have also shown that lanthanum and verapamil can block the potential-dependent and sodium-dependent movements of calcium across the membrane of isolated brain synaptosomes (Erdreich *et al.*, 1983; Gill *et al.*, 1981; Nachshen & Blaustein, 1979). We therefore examined whether in addition to verapamil, the other compounds, screened in this study for synergism to doxorubicin, can also block the calcium uptake into isolated synaptosomes. The inhibition of the sodium-dependent and membrane potential-dependent calcium uptake, obtained by these compounds, is shown in Table III.

An appreciable uptake of calcium could not be obtained in sodium phosphate or potassium phosphate loaded membrane vesicles prepared from either cell line.

### Discussion

Dopyridamole was found in the present study to be highly potent in restoring the sensitivity of P388/ADR cells to doxorubicin (Figures 1 and 2). In previous studies, some of the pharmacological activities of dipyridamole were related to its ability to block adenosine uptake (Liu & Feinberg, 1973; Born & Mills, 1969). Inhibition of adenosine uptake was also recently reported for phenothiazines like thioridazine (Phillis & Wu, 1981). However, papaverine and nitrobenzylthioinosine, which are also effective inhibitors of adenosine uptake (Born & Mills, 1969; Lauzon & Paterson, 1977), do not have doxorubicin synergistic activity (Table I and Ramu *et al.*, 1984*a*). We therefore suggest that the doxorubicin synergistic effect of dipyridamole is not related to its ability to block adenosine transport.

The data presented in this study (Tables I and II) indicate that dipyridamole, verapamil and nicardipine have similar potencies in restoring the sensitivity of P388/ADR cells to doxorubicin. Previous experiments in excitable tissues have demonstrated that verapamil and nicardipine can block the cell membrane calcium channels (Triggle, 1982). However, such an activity could not be demonstrated for dipyridamole (Table III and Mustafa & Nakagawa, 1983). The inability of dipyridamole to block the sodium-dependent or the membrane potential-dependent calcium uptake in synaptic plasma membrane vesicles suggests that the synergism of these compounds with doxorubicin in the drug-resistant P388 cells is not related to calcium channel blocking activity. This suggestion is further supported by the findings that in plasma membrane vesicles, prepared from either P388 or P388/ADR cells, no sodium-dependent or membrane potential-dependent calcium uptake could be demonstrated. Furthermore, Toll (1982) has recently demonstrated that the calcium channel blocking activity in exitable membranes, by calcium

**Table III** Inhibition of sodium-dependent (A) and membrane dependent (B) calcium uptake in synaptic plasma membrane vesicles

| | *Concentration* | % *Inhibition of A* | % *Inhibition of B* |
|---|---|---|---|
| Lanthanum | 5 μM | 19.9 | 0 |
| Lanthanum | 50 μM | 83.4 | 58.0 |
| Perhexiline | 100 μM | 87.1 | 73.7 |
| Chlorocyclizine | 100 μM | 55.5 | 48.5 |
| Chloroquine | 100 μM | 44.6 | 49.6 |
| Phentolamine | 100 μM | 35.1 | 39.0 |
| Guanethidine | 100 μM | 36.4 | 42.4 |
| Verapamil | 100 μM | 25.8 | 19.4 |
| Dipyridamole | 100 μM | 0 | 0 |

antagonists, was related to their ability to inhibit the high affinity binding of [$^3$H]-Nitrendipine (another calcium antagonist) to these membranes. However, specific calcium-dependent binding of [$^3$H]-Nitrendipine could not be demonstrated in membranes prepared from either P388 or P388/ADR cells (Dr R. Fine, Personal Communication). Also, if the doxorubicin synergistic effect of the compounds tested was indeed related to their blocking activity of calcium channels, one would also expect that their potency as doxorubicin synergists would be in the following order: lanthanum > perhexilin > chlorcyclizine > chloroquine > phentolamine > guanethidine > verapamil (Table III). However, the data presented in Tables I and II and in our previous study (Ramu *et al.*, 1984*b*) suggest that this is not the case. This lack of correlation can also be demonstrated by comparing the doxorubicin synergistic activity of the so called calcium channel antagonists (Tsuruo *et al.*, 1983*b*), with their activity in blocking calcium channels (Fleckenstein, 1977; Lee & Tsien, 1983; Triggle, 1981, 1982).

As is shown in Tables I and II, nifedipine, unlike its structural analogue, nicardipine, has only a minimal doxorubicin synergistic activity. Similar results were obtained by others (Table III in Tsuruo *et al.*, 1983*a*). It is therefore suggested that the 2-(N-benzyl)-N-methylamino-ethyl moiety of the nicardipine is important for the doxorubicin synergistic activity of this drug. A similar structure can be found in meclizine, verapamil and more remotely in dipyridamol, perhexiline, chlorcyclizine, thioridazine, and MDL 6792.

In the present study, thioridazine was found to have a moderate synergism to doxorubicin in the P388/ADR cell line. Similar results were obtained with other phenothiazines (Tsuruo *et al.*, 1982; Ganapathi & Grabowski, 1983; Inaba *et al.*, 1981). Recently, Kauffman & Conery (1983) have demonstrated that thioridazine and some other phenothiazines were effective inhibitors of the binding of [$^3$H]-Nitrendipine to cardiac muscle cell membranes. However, as previously discussed, this characteristic does not seem related to the restoration of sensitivity to doxorubicin in drug-resistant cells. There were also suggestions that the phenothiazines exert some of their pharmacological effects by blocking the calmodulin mediated activities (Weiss *et al.*, 1980). Subsequently it was suggested that the inhibition of the action of calmodulin is related to the synergism of these compounds with doxorubicin (Tsuruo *et al.*, 1982). In fact the ability to bind calmodulin and/or block its activities was also shown for some other doxorubicin synergistic compounds like verapamil, prenylamine, dilthiazem, nicardipine, nimodipine, dibucaine, propranolol and phentolamine (Johnson, 1983*a,b*; Epstein *et al.*, 1982; Tsuruo *et al.*, 1982; Volpi *et al.*, 1981; Earl *et al.*, 1982). However, as in the case of calcium channel blockade, there is no correlation between the potencies of these drugs in inhibiting calmodulin-mediated effects and their activity in restoring the sensitivity of drug-resistant cells to doxorubicin. These and other drugs which were shown to antagonize calmodulin-induced activities are of a wide range of chemical classes and have a wide spectrum of pharmacological activities (Vincenzi, 1982). It was suggested that a feature common to all these agents is that they are amphipathic and cationic at physiological pH, and that their binding to calmodulin is not particularly specific (Vincenzi, 1981). These amphipathic cationic compounds can share other activities that are not calmodulin dependent (Vincenzi *et al.*, 1982). More relevant, perhaps, is the recent observation that the calmodulin inhibitory activity of many compounds is related to their ability to stabilize the erythrocyte membrane (Bereza *et al.*, 1982). It is therefore suggested that the doxorubicin synergistic activity of these compounds is related to their interaction with the cell membrane rather than to calcium channel blockade or to inhibition of calmodulin mediated drug efflux. In our previous studies (Ramu *et al.*, 1983; 1984*c*), major differences were found in the characteristics of the lipid domains of the plasma membrane of P388/ADR cells compared to those of the parent P388 cell line. Therefore the preferential enhancement, by these compounds, of the doxorubicin cytotoxicity in P388/ADR cells, may further indicate that specific interaction with the cell membrane lipid domain, is related to the doxorubicin synergistic activity.

The present results indicate that certain drugs restore the effectiveness of doxorubicin against resistant cells *in vitro*. They imply that concomitant administration of these drugs in patients may result in enhanced chemotherapeutic activity of doxorubicin in refractory patients. The finding that the increase in doxorubicin potency was not observed in drug sensitive cells suggest that the synergism may be limited to the drug-resistant cells. However, prior to clinical trials, *in vitro* studies demonstrating no decrease in the therapeutic index of doxorubicin in the presence of these drugs are needed.

This work was supported by grant from the Israel Cancer Research Fund (to A.R.). We thank Shela Rosenberg for excellent technical assistance.

### References

BEREZA, U.L., BREWER, G.J. & MIZUKAMI, I. (1982). Association of calmodulin inhibition, erythrocyte membrane stabilization and pharmacological effects of drugs. *Biochim. Biophys. Acta*, **692**, 305.

BORN, G.V.R. & MILLS, D.C.B. (1969). Potentiation of the inhibitory effect of adenosine on platelet aggregation by drugs that prevent its uptake. *J. Physiol. London*, **202**, 41P.

BOWN, D. & GOLDMAN, I.D. (1975). The relationship among transport, intracellular binding, and inhibition of RNA synthesis by actinomycin D in Ehrlich ascites tumor cells in vitro. *Cancer Res.*, **35**, 3054.

CARLSEN, S.A., TILL, J.E. & LING, V. (1976). Modulation of membrane drug permeability in Chinese Hamster ovary cells. *Biochim. Biophys. Acta*, **455**, 900.

CHLEBOWSKI, R.T., BLOCK, J.B., CUNDIFF, D. & DIETRICH, F. (1982). Doxorubicin cytotoxicity enhanced by local anesthetics in a human melanoma cell line. *Cancer Treat. Rep.*, **66**, 121.

EARL, C.Q., PROZIALECK, W.C. & WEISS, B. (1982). Inhibition of calmodulin activity by alpha adrenergic antagonists. *Fed. Proc.*, **41**, 1565.

EPSTEIN, P.M., FISS, K., HACHISU, R. & ANDRENYAK, D.M. (1982). Interaction of calcium antagonists with cyclic AMP phosphodiesterases and calmodulin. *Biochem. Biophys. Res. Commun.*, **105**, 1142.

ERDREICH, A., SPANIER, R. & RAHAMIMOFF, H. (1983). The inhibition of Na-dependent Ca uptake by verapamil in synaptic plasma membrane vesicles. *Eur. J. Pharmacol.*, **90**, 193.

FLECKENSTEIN, A. (1977). Specific pharmacology of calcium in myocardium, cardiac pacemakers and vascular smooth muscle. *Ann. Rev. Pharmacol. Toxicol.*, **17**, 149.

GANAPATHI, R. & GRABOWSKI, D. (1983). Enhancement of sensitivity to adriamycin in resistant P388 leukemia by the calmodulin inhibitor trifluoperazine. *Cancer Res.*, **43**, 3696.

GILL, D.L., GROLLMAN, E.F. & KOHN, L.D. (1981). Calcium transport mechanisms in membrane vesicles from Guinea Pig brain synaptosomes. *J. Biol. Chem.*, **256**, 184.

INABA, M., FUJIKURA, R., TSUKAGOSHI, S. & SAKURAI, Y. (1981). Restored *in vitro* sensitivity of adriamycin- and vincristine-resistent P388 leukaemia with reserpine. *Biochem. Pharmacol.*, **30**, 2191.

JOHNSON, J.D. (1983*a*). Interaction of hydrophobic inhibitory ligands with calmodulin. *Biophys. J.*, **41**, 306a.

JOHNSON, J.D. (1983*b*). Allosteric interactions among drug binding sites on calmodulin. *Biochem. Biophys. Res. Commun.*, **112**, 787.

KAUFFMAN, R.F. & CONERY, B.G. (1983). Inhibition of [$^3$]Nitrendipine binding to cardiac membranes by calmodulin antagonists. *Fed. Proc.*, **42**, 573.

KLEIN, M.E. & FRAYER, K. (1978). Alteration in secondary adriamycin resistance by amphotericin and hyperthermia. *Proc. Am. Ass. Cancer Res.*, **19**, 84.

LAUZON, G.J. & PATERSON, A.R.P. (1977). Binding of the nucleoside transport inhibitor nitrobenzylthioinosine to HeLa cells. *Mol. Pharmacol.*, **13**, 883.

LEE, K.S. & TSIEN, R.W. (1983). Mechanism of calcium channel blockade by verapamil, D600, Diltiazem and nitrendipine in single dialysed heart cells. *Nature*, **302**, 790.

LIU, M-S. & FEINBERG, H. (1973). Effect of persantin on nucleoside metabolism of the perfused rabbit heart. *Biochem. Pharmacol.*, **22**, 1181.

MEDOFF, J., MEDOFF, G., GOLDSTEIN, M.N., SCHLESSINGER, D. & KOBAYASHI, G.S. (1975). Amphotericin B-induced sensitivity to actinomycin D in drug-resistant HeLa cells. *Cancer Res.*, **35**, 2548.

MIZUNO, S. & ISHIDA, A. (1982*a*). Potentiation of bleomycin cytotoxicity by membrane-interacting drugs and increased calcium ions. *Biochem. Biophys. Res. Commun.*, **107**, 1021.

MIZUNO, S. & ISHIDA, A. (1982*b*). Selective enhancement of the cytotoxicity of the bleomycin derivative, peplomycin, by local anesthetics alone and combination with hyperthermia. *Cancer Res.*, **42**, 4726.

MIZUNO, S. & ISHIDA, A. (1982*c*). Selective enhancement of bleomycin cytotoxicity by local anesthetics. *Biochem. Biophys. Res. Commun.*, **105**, 425.

MUSTAFA, S.J. & NAKAGAWA, Y. (1983). Calcium blocking activity of dilazep, lidoflazine, dipyridamole and adenosine in comparison to nifedipine and verapamil in dog coronary artery. *Blood Vess.*, **20**, 203.

NACHSHEN, D.A. & BLAUSTEIN, M.P. (1979). The effects of some organic "calcium antagonists" on calcium influx in presynaptic nerve terminals. *Mol. Pharmacol.*, **16**, 579.

OZOLS, R.F., HOGAN, W.M., GROTZINGER, K.R., McCOY, W. & YOUNG, R.C. (1983). Effects of amphotericin B on adriamycin and melphalan cytotoxicity in human and murine ovarian carcinoma and in L1210 leukemia. *Cancer Res.*, **43**, 959.

PHILLIS, J.W. & WU, P.H. (1981). Phenothiazines inhibit adenosine uptake by rat brain synaptosomes. *Can. J. Physiol. Pharmacol.*, **59**, 1108.

RAHAMIMOFF, H. & SPANIER, R. (1979). Sodium-dependent calcium uptake in membrane vesicles derived from rat brain synaptosomes. *FEBS Let.*, **104**, 111.

RAMU, A., GLAUBIGER, D., MAGRATH, I.T. & JOSHI, A. (1983). Plasma membrane lipid structural order in doxorubicin-sensitive and -resistant P388 cells. *Cancer Res.*, **43**, 5533.

RAMU, A., GLAUBIGER, D., SOPREY, P., REAMAN, G.H. & FEUERSTEIN, N. (1984*a*). 5′-Nucleotidase activity and arachidonate metabolism in doxorubicin sensitive and resistant P388 cells. *Br. J. Cancer*, **49**, 447.

RAMU, A., FUKS, Z., GATT, S. & GLAUBIGER, D. (1984*b*). Reversal of acquired resistance to doxorubicin in P388 murine leukemia cells by perhexiline maleate. *Cancer Res.*, **44**, 144.

RAMU, A., GLAUBIGER, D. & WEINTRAUB, H. (1984*c*). Differences in lipid composition of doxorubicin-sensitive and -resistant P388 cells. *Cancer Treat. Rep.*, **68**, 637.

REUTER, H. (1983). Calcium channel modulation by neurotransmitters, enzymes and drugs. *Nature*, **301**, 569.

RIEHM, H. & BIEDLER, J.L. (1972). Potentiation of drug effect by tween 80 in Chinese Hamster cells resistant to actinomycin D and daunomycin. *Cancer Res.*, **32**, 1195.

SEEBER, S., OSIEKA, R., SCHMIDT, C.G., ACHTERRATH, W. & CROOKE, S.T. (1982). *In vivo* resistance towards anthracyclines, etoposide and cisdiamminedichloplatinum (11). *Cancer Res.*, **42**, 4719.

TOLL, L. (1982). Calcium antagonists. High-affinity binding and inhibition of calcium transport in a clonal cell line. *J. Biol. Chem.*, **257**, 13189.

TRIGGLE, D.J. (1981). Calcium antagonists: Basic chemical and pharmacological aspects. In: *New Perspectives on Calcium Antagonists*. p. 1. (Ed. Weiss, Bethesda, American Physiological Society.

TRIGGLE, D.J. (1982). Biochemical pharmacology of calcium blockers. In: *Calcium Blockers, Mechanisms off Action and Clinical Applications*. p. 121 (Eds. Flaim & Zelis) Baltimore, Urban & Schwarzenberg.

TSURUO, T., IIDA, H., TSUKAGOSHI, S. & SAKURAI, Y. (1982). Increased accumulation of vincristine and adriamycin in drug-resistant P388 tumour cells following incubation with calcium antagonists and calmodulin inhibitors. *Cancer Res.*, **42**, 4730.

TSURUO, T., IIDA, H., NOJIRI, M., TSUKAGOSHI, S. & SAKURAI, Y. (1983*a*). Circumvention of vincristine and adriamycin resistance *in vitro* and *in vivo* by calcium influx blockers. *Cancer Res.*, **43**, 2905.

TSURUO, T., IIDA, H., TSUKAGOSHI, S. & SAKURAI, Y. (1983*b*). Potentiation of vincristine and adriamycin effects in human hemopoietic tumor cell lines by calcium antagonists and calmodulin inhibitors. *Cancer Res.*, **43**, 2267.

VALEROITE, F., MEDOFF, G. & DIECKMAN, J. (1979). Potentiation of anticancer agent cytotoxicity against sensitive and resistant AKR leukemia by amphotericin B. *Cancer Res.*, **39**, 2041.

VINCENZI, F.F. (1981). Calmodulin pharmacology. *Cell Calcium*, **2**, 387.

VINCENZI, F.F. (1982). The pharmacology of calmodulin antagonism: A reappraisal. In: *Calmodulin and Intracellular $Ca^{++}$ Receptors*. p. 1. (Eds. Kakiuchi *et al.*) New York, Plenum Press.

VINCENZI, F.F., ADUNYAH, E.S., NIGGLI, V. & CARAFOLI, E. (1982). Purified red blood cell $Ca^{++}$-pump ATPase: Evidence for direct inhibition by presumed anti-calmodulin drugs in the absence of calmodulin. *Cell Calcium*, **3**, 545.

VOLPI, M., SHA'AFI, R.I., EPSTEIN, P.M., ANDRENYAK, D.M. & FEINSTEIN, M.B. (1981). Local anesthetics, mepacrine and propranolol are antagonists of calmodulin. *Proc. Natl Acad Sci.*, **78**, 795.